# Nail Manifestations in COVID-19: Insight into a Systemic Viral Disease

**DOI:** 10.1159/000518087

**Published:** 2021-08-17

**Authors:** Ana Preda-Naumescu, Kayla Penney, Ross L. Pearlman, Robert T. Brodell, Carlton Ralph Daniel, Vinayak K. Nahar

**Affiliations:** ^a^University of Alabama at Birmingham School of Medicine, Birmingham, Alabama, USA; ^b^LSU Health Shreveport School of Medicine, Shreveport, Louisiana, USA; ^c^Department of Dermatology, School of Medicine, University of Mississippi Medical Center, Jackson, Mississippi, USA; ^d^Department of Preventive Medicine, School of Medicine/John D. Bower School of Population Health, University of Mississippi Medical Center, Jackson, Mississippi, USA

**Keywords:** SARS-CoV-2, Nails, Red half-moon sign, Transverse orange nail lesions, Mees' lines, Beau's lines, Periungual pernio-like changes

## Abstract

Nail manifestations are 1 of the several extrapulmonary findings associated with COVID-19 caused by the severe acute respiratory syndrome coronavirus 2 (SARS-CoV-2). Nail changes, however, have been largely ignored and not yet summarized. This article is intended to increase awareness of nail manifestations of SARS-CoV-2, which occur weeks to months after acute infection and the periungual pernio-like changes may occur concomitantly with infection. An electronic search was carried out in PubMed (Medline), Science Direct, and Scopus databases. The following keywords and all of their possible combinations were used to identify studies: “SARS-CoV-2,” “COVID-19,” “Coronavirus,” “2019-ncov,” “nail,” and “nails.” Six case reports were included in this study. Manifestations identified included red half-moon sign, transverse orange nail lesions, Mees' lines, and Beau's lines. Though largely nonspecific, these findings can be recognized with the onset of symptom onset or as late as 16 weeks following the disease. Some of these findings are shared with other conditions associated with a proinflammatory state. Nail changes offer unique insight into the pathophysiologic basis for SARS-CoV-2 and they may serve as diagnostic clues.

## Introduction

COVID-19 is the contagious respiratory tract disease caused by severe acute respiratory syndrome coronavirus 2 (SARS-CoV-2). It became a household name in spring of 2020 when the World Health Organization declared a world-wide pandemic [1]. Since the first outbreak was reported in Wuhan, China, in December of 2019, >132 million global cases have been confirmed with over 2.8 million global deaths as of April 7, 2021 [2]. Cutaneous findings vary with patient demographics and the timing of infection. They are classified as erythema-edema with vesicles or pustules (pseudo-chilblain), other vesicular eruptions, urticarial lesions, maculopapular eruptions, and livedo or necrosis [3].

Because some nail manifestations of SARS-CoV-2 may occur months after the acute infection, they have been largely overlooked. Nonspecific ungual changes associated with a variety of infectious diseases have been described and may offer insight into the more serious systemic manifestations of these conditions [4]. This article highlights these findings and the unique nail lesions that have been described following infection with the novel coronavirus.

## Methods

### Eligibility Criteria

Case reports were considered for inclusion based on the following criteria if: (1) nail changes in COVID-19 were assessed; (2) publication occurred in peer-reviewed journals; and (3) writing was in English. Articles were excluded based on the following criteria: (1) duplicates; (2) information appeared as conference abstracts, opinion, editorials, perspective, viewpoints, news, letters to the editor, commentaries, reviews, feature articles, white papers, and guidelines; (3) studies were incomplete or ongoing; and (4) unpublished articles.

### Literature Search Strategy

A literature review analyzed currently available published cases of documented SARS-CoV-2 with nail manifestations. An electronic search was carried out in PubMed (Medline), Science Direct, and Scopus databases from December 1, 2019, to April 2, 2021. To identify the articles, possible combinations of the following keywords were utilized: “SARS-CoV-2,” “COVID-19,” “Coronavirus,” “2019-ncov,” “nail,” and “nails.” Additional searches in the Google Scholar were performed to ensure that no studies were overlooked.

### Search Outcome

Two independent investigators performed the search and reviewed the titles, abstracts, full texts to determine if they met the inclusion criteria. A total of 1,094 studies were identified using the electronic search processes. After removing duplicates, the titles and abstracts of 56 articles were reviewed, and then 14 articles were left for full-text review. During full-text review, 8 articles were omitted based on the inclusion criterion, and 6 articles met the predetermined eligibility requirements. Table 1 provides a brief overview of the COVID-19 cases with nail manifestations focusing on clinical presentation, location, and temporal relationship to viral infection. The remainder of the review provides more detailed insight to these unique nail presentations in hopes of helping health care providers recognize signs of this systemic disease.

## Results

### The Red Half-Moon Sign

**Fig. 1:**
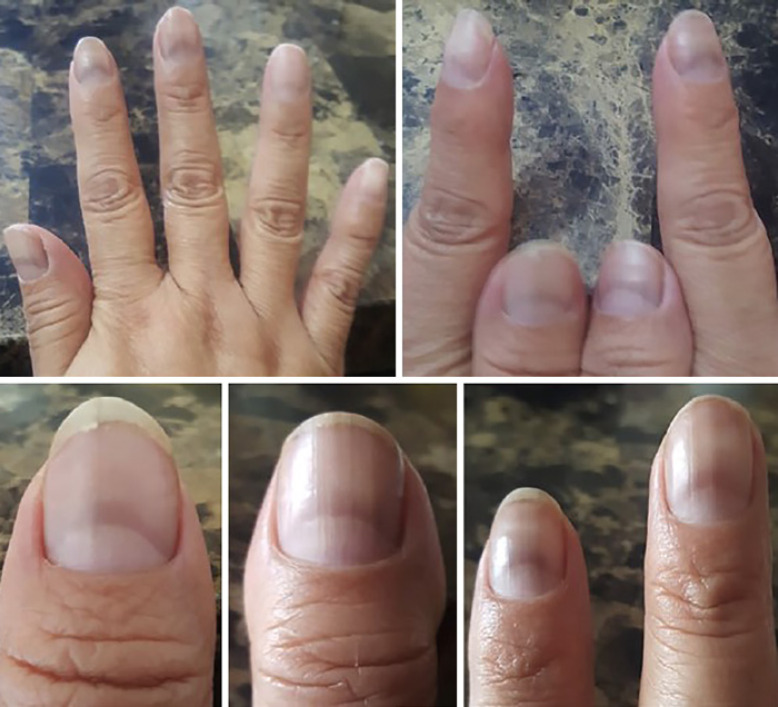
The red half-moon sign. Red, horizontally oriented, convex bands span the distal margin of the lunula 2 days after the onset of SARS-CoV-2 infection. Permission to use this image was granted by the International Journal of Dermatology on April 23, 2021. Fig. 1, page 1414 [6]. SARS-CoV-2, severe acute respiratory syndrome coronavirus 2.

The red half-moon sign is a novel manifestation of coronavirus infection. It has been described as appearing following symptom onset. Presentation includes appearance of a distally convex half-moon shaped red band surrounding the distal margin of the lunula, which may appear on all fingernails [5]. The affected patient denied associated symptoms and had no other cutaneous manifestations of COVID-19 [5]. This novel finding was corroborated in a second patient 2 days following onset of COVID-19 symptoms (Fig. 1) [6]. These clinical findings may represent microvascular injury of the capillary network of the distal subungual arcade secondary inflammatory immune response and a procoagulant mileu that has been previously associated with SARS-CoV-2 infection [5]. Biopsies were not performed in either case. Similarly, transverse nail-bed lines have been previously reported in association with inflammatory conditions such as Kawasaki disease [5]. Also appearing similar to the above findings, erythematous periungual findings known as “pernio-like” have been found in inflammatory states that are frequently seen in various systemic diseases [7].

### Transverse Orange Nail Lesions

**Fig. 2:**
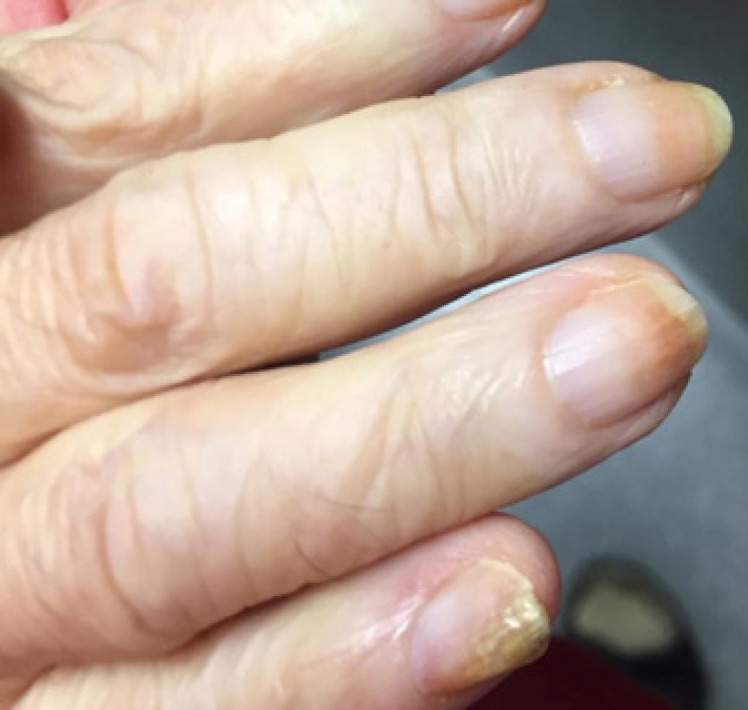
Transverse orange nails. Distal orange discoloration appeared 16 weeks after SARS-CoV-2 infection with a straight border separating this finding from the proximal healthy-appearing nail bed. Permission to use this image was granted by Dermatologic Therapy on April 23, 2021. Fig. 1, page 1 [7]. SARS-CoV-2, severe acute respiratory syndrome coronavirus 2.

Orange discolorations at the end of the fingernail beds were noted in an elderly, female patient 16 weeks after the onset of COVID-19 symptoms [8]. Physical exam revealed orange discoloration of the distal nail plate with a demarcation separating them from the healthy-appearing nail bed areas (Fig. 2) [8]. The shape of the proximal border of the discoloration followed the shape of the nail lunula which is consistent with a systemic cause of this finding [8]. A positive PCR diagnosis and IgG against SARS-CoV-2 confirmed the diagnosis. The nail findings were unchanged 1 month later [8]. The patient also had ferropenic anemia [8]. The authors noted similar findings in the nails of Kawasaki patients, once more alluding to the possibility of complement-medicated microvascular injury as the potential explanation behind this phenomenon [7]. The significance of the low serum iron levels is unknown, but this finding has been associated with more severe SARS-CoV-2 disease [8].

A significant onychopathological indicator of chronic renal failure, Lindsay's nail, or the half-and-half nail can resemble this finding. The key to distinguishing between these phenomena may be the degree of orange discoloration that is found in COVID affected nails [9]. Furthermore, this finding may also be confused for pseudo half-and-half nails, a similar finding seen in patients with psoriasis [9].

### Transverse Leukonychia (Mees' Lines)

**Fig. 3:**
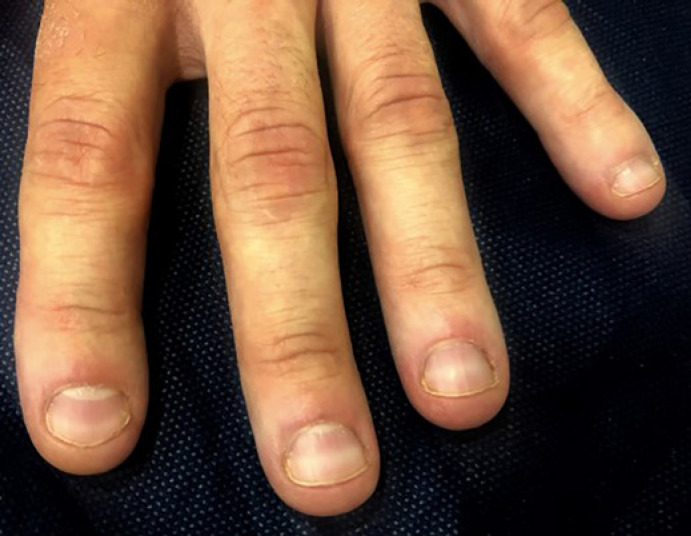
Transverse leukonychia (Mees' lines). Transverse, nonblanchable white lines are present on all fingernails that slowly grew out. This photograph was taken 45 days after the lines were first noted during a hospitalization for SARS-CoV-2 infection. Permission to use this image was granted by Dermatologic Therapy on April 23, 2021. Fig. 1, page 5 [9]. SARS-CoV-2, severe acute respiratory syndrome coronavirus 2.

Transverse leukonychia or Mees' lines are transverse white lines in the finger or toe nails. These exam findings have been previously described in association with numerous systemic disorders including arsenic poisoning due to deposition of arsenic in keratin rich tissues [4, 10]. Spanning the width of the nail plate, Mees' lines often affect all fingernails and take 3–6 weeks to develop [4, 10]. Transverse leukonychia was recently described in all ten fingernails in a previously healthy 57-year-old Spanish male with SARS-CoV-2 bilateral pneumonia confirmed by PCR [11]. The patient was treated with lopinavir/ritonavir 100 mg/400 mg bid for 10 days with adequate response [5]. The transverse, nonblanchable white lines progressively migrated with the growth of the nail and were present 45 days later (Fig. 3) [11].

Mees' lines are also associated with a number of other systemic stressors including acute renal failure, congestive heart failure, ulcerative colitis, cancer, infections, systemic lupus erythematous, and the use of chemotherapeutic agents [11]. The lines appear to be the product of altered keratinization of the nail plate that occurs as systemic conditions induce temporary dysfunction of nail growth [11]. Importantly, they are nonblanchable which helps distinguish them from Muehrcke lines, which are associated with hypoalbuminemia and altered nail bed vascularization [11].

### Beau's Lines

**Fig. 4:**
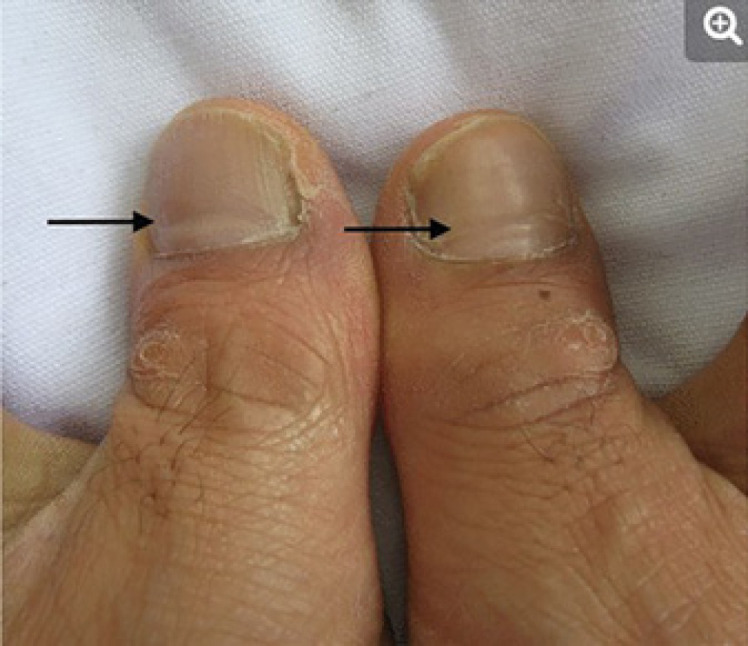
Beau's lines. Horizontal nail grooves were noted on all ten fingernails growing distally with nail growth. This photograph was taken 3 and a half months following a diagnosis of SARS-CoV-2 [10]. Permission to use this image was granted by Canadian Medical Association Journal on April 23, 2021. Fig. 1, page E1040 [10]. SARS-CoV-2, severe acute respiratory syndrome coronavirus 2.

Beau's lines are transverse grooves in the nail plate caused by temporary diminution or suspension of nail growth approximately 2–3 weeks following acute stress to the nail matrix [4, 11]. These lines appear as the involved nail emerges from the proximal nail fold [6]. Beau's lines have been described following local trauma to the nail as well as systemic illnesses, severe malnutrition, autoimmune conditions, including pemphigus and Raynaud disease, Kawasaki disease, and chemotherapy [12]. A 45-year-old man recently presented with horizontal nail grooves across the proximal nail folds of both finger and toe nails approximately 3 and a half months following a diagnosis of SARS-CoV-2 (Fig. 4) [12]. In another report, sunken, white horizontal lines developed in the nails of a 68-year-old Japanese man 1-month following hospital discharge for SARS-CoV-2 infection [13]. The nail findings were clinically defined as leukonychia and Beau's lines with periungual desquamation, a finding well-described in pediatric inflammatory multisystem syndrome [13]. No specific intervention is required for Beau's lines to resolve with continued nail growth.

## Discussion

Manifestations of COVID-19 infection in the nail unit are mostly non-specific possibly caused by the sensitive nature of the nail matrix when impacted by trauma, inflammation, and hypercoagulability. This is certainly the case with Beau's lines which are well described in association with coxsackievirus in addition to COVID-19 [14]. Development of Beau's lines after use of a tourniquet on the upper extremity for hand surgery supports the hypothesis that nail matrix arrest may be secondary to ischemia [15]. Mees' lines may similarly result from the dysregulation of cell turnover at the matrix due to digital ischemia resulting from sepsis and hypotension, microvascular damage, or a hypercoagulable state resulting in abnormal keratinization [16].

The red-half-moon nail sign is exceptional in that it may represent a pathognomonic nail finding in CO­VID-19 infection [5]. These isolated case reports, however, are not yet sufficient to establish this finding as a unique characteristic of COVID-19 infection. COVID “pernio” accounts for 2/3 of skin findings in patients with COVID-19 and is associated with a lymphocytic vasculitis of small dermal vessels with fibrin thrombi [17]. Similarly, complement-mediated vascular injury results in thrombus formation in small vessels in lung tissue, and complement binding to COVID-19 spike glycoproteins has been identified in purpuric skin lesions of COVID-19 patients [18]. Neri et al. [5] have suggested the same mechanism for erythronychia: inflammation and a procoagulant milieu produce microvascular injury of the nail bed.

Nail changes along with cutaneous manifestations may provide valuable insight into underlying systemic manifestations of SARS-CoV-2. Clinicians should continue to document these changes since they may become important diagnostic clues and help clinicians better understand the pathophysiologic basis for this viral illness.

## Conflict of Interest Statement

Robert T. Brodell has participated in multi-center clinical trials with Corevitas (Formerly Corrona) Psoriasis Registry and Novartis. He is also an associate editor of the Journal of the American Academy of Dermatology, Faculty advisor for the American Medical Student Research Journal, and editor-in-chief of Practice Update: Dermatology, and serves as Staff Dermatologist at the GV (Sonny) MONTGOMERY VA HOSPITAL in Jackson, MS. Daniel III C. Ralph is among the board of directors of Council for Nail Disorders, European Nail Society, and St. Dominic Health Services Foundation. He is also Clinical Professor of Dermatology at the University of Alabama at Birmingham. He is also American Dermatological Association Chairman of Endowment Committee. He serves on the editorial board of the Skin Appendages Disorders and on the advisory board of Ortho Pharmaceutical. He is also the co-editor of a book Scher and Daniel's Nails, Fourth edition, Springer, Philadelphia, 2018. He is also a stakeholder of Medimetriks. Ana Preda-Naumescu, Kayla Penney, Ross L. Pearlman, and Vinayak K. Nahar have no conflicts of interest.

## Funding Sources

None declared.

## Author Contributions

A.P.-N., K.P., and V.K.N. contributed to conception and design; A.P.-N., K.P., and V.K.N. contributed to the identification of studies. A.P-N., K.P., and V.K.N. contributed to the extraction of data; all authors contributed to the analysis of data; all authors contributed to the interpretation of data; all authors drafted the article or revised it critically for important intellectual content; all authors gave final approval of the version of the article to be published; all authors agree to be accountable for all aspects of the work in ensuring that questions related to the accuracy or integrity of any part of the work are appropriately investigated and resolved; and all authors have read and approved the manuscript.

## Figures and Tables

**Table 1:** A summary of the reviewed case reports

Author	Condition	Description	Relation to COVID-19	Other
**Neri et al. [5]**	**The red half-moon sign**	**Red convex lesion at the distal margin of the lunula on the fingernail beds**	**Two weeks after symptoms with confirmed diagnosis**	**Reported widening of bands 1 month after appearance**

**Méndez-Flores et al. [6]**	**The red half-moon sign**	**Red convex lesion at the distal margin of the lunula on the fingernail beds**	**Two days after symptoms with confirmed diagnosis**	**Resolved within 1 week**

**Tammaro et al. [8]**	**Transverse orange nails**	**Orange discolorations at the distal tips of the fingernail beds**	**Sixteen weeks after symptoms, appearing simultaneously with a positive IgG test for SARS-CoV-2**	**Remained unchanged at 1 month**

**Fernandez-Nieto et al. [11]**	**Transverse leukonychia (Mees' lines)**	**White, transverse lines across the fingernail beds**	**Appeared concurrently with symptoms and confirmed diagnosis**	**Still present after 45 days, but diminishing as nail growth ensued**

**Alobaida et al. [12]**	**Beau's lines**	**Transverse grooves along the proximal finger and toenail beds**	**Three and a half months after symptoms and confirmed diagnosis**	

**Ide et al. [13]**	**Beau's lines**	**Transverse grooves along the proximal fingernail beds**	**One month after symptoms and confirmed diagnosis**	**Associated with leukonychia and periungual desquamation**

SARS-CoV-2, severe acute respiratory syndrome coronavirus 2.
